# Blood pressure self-monitoring in pregnancy (BuMP) feasibility study; a qualitative analysis of women’s experiences of self-monitoring

**DOI:** 10.1186/s12884-017-1592-1

**Published:** 2017-12-19

**Authors:** Lisa Hinton, Katherine L. Tucker, Sheila M. Greenfield, James A. Hodgkinson, Lucy Mackillop, Christine McCourt, Trisha Carver, Carole Crawford, Margaret Glogowska, Louise Locock, Mary Selwood, Kathryn S. Taylor, Richard J. McManus

**Affiliations:** 1 Nuffield Department of Primary Care Health Sciences, University of Oxford, New Radcliffe House, Woodstock Rd, Oxford, OX2 6GG UK; 2 Institute of Applied Health Research, University of Birmingham, Edgbaston, Birmingham, B15 2TT UK; 3 Oxford University Hospitals NHS Trust, Women’s Centre, John Radcliffe Hospital, Oxford, OX3 9DU UK; 4 Oxford NIHR Biomedical Research Centre, The Joint Research Office, The Churchill Hospital, Block 60, Old Road, Headington, Oxford, OX3 7LE UK; 5 City, University of London, Northampton Square, London, EC1V 0HB UK; 6 Health Services Research Unit, University of Aberdeen, Aberdeen, AB25 2ZD UK

**Keywords:** Pregnancy, Blood pressure, Self-monitoring, Hypertension, Pre-eclampsia, Qualitative, Women’s experiences

## Abstract

**Background:**

Hypertensive disorders in pregnancy are a leading cause of maternal and fetal morbidity worldwide. Raised blood pressure (BP) affects 10% of pregnancies worldwide, of which almost half develop pre-eclampsia. The proportion of pregnant women who have risk factors for pre-eclampsia (such as pre-existing hypertension, obesity and advanced maternal age) is increasing. Pre-eclampsia can manifest itself before women experience symptoms and can develop between antenatal visits. Incentives to improve early detection of gestational hypertensive disorders are therefore strong and self-monitoring of blood pressure (SMBP) in pregnancy might be one means to achieve this, whilst improving women’s involvement in antenatal care. The Blood Pressure Self-Monitoring in Pregnancy (BuMP) study aimed to evaluate the feasibility and acceptability of SMBP in pregnancy.

**Methods:**

To understand women’s experiences of SMBP during pregnancy, we undertook a qualitative study embedded within the BuMP observational feasibility study. Women who were at higher risk of developing hypertension and/or pre-eclampsia were invited to take part in a study using SMBP and also invited to take part in an interview. Semi-structured interviews were conducted at the women’s homes in Oxfordshire and Birmingham with women who were self-monitoring their BP as part of the BuMP feasibility study in 2014. Interviews were conducted by a qualitative researcher and transcribed verbatim. A framework approach was used for analysis.

**Results:**

Fifteen women agreed to be interviewed. Respondents reported general willingness to engage with monitoring their own BP, feeling that it could reduce anxiety around their health during pregnancy, particularly if they had previous experience of raised BP or pre-eclampsia. They felt able to incorporate self-monitoring into their weekly routines, although this was harder post-partum. Self-monitoring of BP made them more aware of the risks of hypertension and pre-eclampsia in pregnancy. Feelings of reassurance and empowerment were commonly reported by the women in our sample.

**Conclusions:**

SMBP in pregnancy was both acceptable and feasible to women in this small pilot study.

## Background

Hypertensive disorders in pregnancy are a leading cause of maternal and fetal morbidity worldwide [1]. Raised blood pressure (BP) affects 10% of pregnancies worldwide, of which almost half develop pre-eclampsia [2]. The proportion of pregnant women who have risk factors for pre-eclampsia (such as pre-existing hypertension, obesity and advanced maternal age) is increasing [3, 4].

Pre-eclampsia can manifest itself before women experience symptoms [5–7] and can develop between antenatal visits. Incentives to improve early detection of gestational hypertensive disorders are therefore strong and self-monitoring of blood pressure (SMBP) in pregnancy might be one means to achieve this, whilst improving women’s involvement in antenatal care.

In the general population, SMBP is increasingly popular with patients, the general public and health professionals. A recent UK study indicated around a third of people with hypertension self-monitor BP [8], which is more accurate for diagnosis and better correlated with cardiovascular outcome than measurements taken in the clinic [9, 10]. However, few data exist regarding SMBP in pregnancy, which leaves many unanswered questions about its acceptability, safety and efficacy: whether women will engage with self-monitoring, whether it will be accurate and affect pregnancy outcomes, and whether it will increase feelings of medicalisation and anxiety for women during their pregnancy [11–14]. Recent research exploring the acceptability and psychological impact of antenatal screening for pre-eclampsia has highlighted the potential positive, negative and unintended effects of providing screening information for pre-eclampsia [15].

The Blood Pressure Self-Monitoring in Pregnancy (BuMP) study aimed to evaluate the feasibility and acceptability of SMBP in pregnancy. This paper reports the qualitative data collected as part of the feasibility study and considers it in the context of the increasing popularity of SMBP in the population at large and the impact it may have on women’s experiences of their pregnancy [8, 16] .

## Methods

Women who were deemed at higher risk of pre-eclampsia (who had any of: previous pre-eclampsia, family history of pre-eclampsia, hypertension, pregnancy interval of 10 years, age 40 or over, high BMI, first or multiple pregnancy, renal disease) according to the National Institute for Care and Excellence (NICE) guidelines [17] were invited at between 12 and 16 weeks in their pregnancy to take part in a study that asked them to monitor their own BP at home, in addition to standard antenatal care through their pregnancy, and for up to 6 weeks post-partum. Participants self-monitored their own blood pressure using an automated electronic sphygmomanometer validated for use in pregnancy and pre-eclampsia (Microlife WatchBP home). The women were asked to self-monitor, taking two measurements in both morning and evening following five minutes rest on 3 days a week (Monday, Wednesday and Friday). Women used a traffic light system (red – high BP, urgent action required; amber – raised BP, action required; green – normal BP) developed from our previous work to classify raised BP and were given written advice regarding actions in the light of persistently high readings [18]. Optionally they could text their readings to a central server and receive automated responses via the NHS “Florence” Simple Telehealth system [19, 20], which also provided text prompts and reminders to the participants.

### Interviews

One hundred and seventy women recruited to the BuMP study provided home readings during their pregnancy. At study appointments, women were invited by the research midwife to take part in an interview study to find out more about their experiences of the pilot study, and their views about SMBP. They were given an information pack that included information about the interview and a reply slip. Fifteen women returned slips (13 from Oxfordshire, 2 from Birmingham) and interviews were conducted postpartum with all the women who volunteered. Interviewee identifiers after the quotations presented here relate to the identifiers in Table 1. Those agreeing to take part in the interviews were representative of the study population, including a range of ethnicity, age, demographic area and risk factors used for inclusion in the study. However, no women who dropped out of the self-monitoring of blood pressure study agreed to be interviewed.

**Table 1:** Qualitative interview sample

Interviewee No	Ethnicity	Age *	Reason/s for inclusion	Diagnosis of Hypertension	IMD^a^	Highest Qualification
1	White British	42	Age, BMI, hypertensive in previous pregnancy, PE in previous pregnancy	No	1	First Degree
2	White British	29	First pregnancy, hypertensive before pregnancy	Yes, CH, GH	7	GCSE, O Level or CSE
3	Pakistani	23	First pregnancy	No	2	First Degree
4	White Other	40	BMI, an inter-pregnancy interval of >10 years previous (previous early miscarriage)	No	5	Professional qualification
5	White British	33	Family history of PE, hypertensive in previous pregnancy	No	9	Post-graduate or above
6	White British	33	First pregnancy	No	5	First degree
7	White British	38	First pregnancy, BMI	No	7	First degree
8	White British	27	First pregnancy, multiple pregnancy	No	8	First degree
9	unknown	31	First pregnancy, BMI	No	1	unknown
10	African	35	BMI	No	5	First degree
11	White British	36	Family history of PE, Previous hypertension, hypertensive in previous pregnancy	Yes, CH	10	Professional qualification
12	White British	30	First pregnancy	No	10	Post-graduate or above
13	White British	34	Hypertension in previous pregnancy	Yes, CH	4	First degree
14	White British	40	Age, first pregnancy	No	7	Post graduate qualification
15	White British	35	BMI, first pregnancy	No	10	Professional qualification

*Age at the time of interview

^a^Index of Multiple Deprivation

IMD – Index of Multiple Deprivation; Department for Communities and Local Government. English indices of deprivation 2015. http://imd-by-postcode.opendatacommunities.org/

*CH* Chronic hypertension, *GH* Gestational hypertension, *PE* Pre-eclampsia

Semi-structured interviews were conducted with the women in their own homes, after they had given birth and stopped monitoring their BP as part of the study. The interviews, conducted by an experienced qualitative researcher with a social science rather than health professional background (LH) were semi-structured and included women’s experiences of pregnancy, their attitudes to self-monitoring and their experiences of participating in the BuMP study. Interviews ranged in length from 30 to 60 min and typically took place between 3 and 6 months after birth in order that women had time recover from the immediate birth experience and to assimilate their pregnancy monitoring experience but that it was still relatively recent, although some maternity research indicates there is little recall bias regarding birth experiences [21]. An interview topic guide was developed, and refined as interviews were collected (see appendix for Topic Guide). Interviews were audio recorded and transcribed for analysis. Initial thematic coding and analysis was undertaken by LH. A framework that included the themes reported here was developed in collaboration with the multidisciplinary study team. A Framework analysis was undertaken, employing five stages (familiarisation with the data, identifying a thematic framework, indexing, charting and finally mapping and interpretation) [22]. Interviewing continued until data saturation was reached for the themes reported in this paper [23]. Framework analysis has been developed specifically for applied or policy relevant qualitative research, and was used in this study so that specific questions could be asked to inform future research [24]. In this study, the framework was not a priori, however, but derived from initial analysis of the interviews. Ethical approval was provided for the interviews as part of the Narratives of health and illness for Healthtalkonline 2012.’ IRAS ID 112111, approved by NRES Committee South Central- Berkshire ref.: 12/ SC/0495.

## Results

### Overview

Of the 15 women interviewed, 10 were in their first pregnancy, five had previous experience of high BP or pre-eclampsia or family history of pre-eclampsia, two had age 40 or over at the time of the study, six had high BMI, one had pregnancy interval of over 10 years and one had multiple pregnancy. None of the women developed pre-eclampsia, although three were diagnosed with hypertension (>140/90 mmHg) (see Table 1).

Women who took part were willing to engage with monitoring their own BP, feeling that it could reduce some of the anxiety around their health during pregnancy. They felt able to incorporate self-monitoring into their weekly routines, although keeping up with the monitoring once they had stopped work and lost that daily routine could be harder and it was particularly hard after the baby was born.

Being involved in the study made women more aware of the risks of raised BP and pre-eclampsia. Many felt reassured and empowered, particularly if they had previous experience of the disease.

### Detailed analysis

The framework analysis interrogated the interview data across five themes which themselves had emerged from early stages of analysis [21].

1. Women’s attitudes to their health
2. SMBP as reassuring not anxiety provoking
3. Understanding of the trial, BP and risks
4. Experiences of pregnancy and identifying raised BP
5. Practicalities of SMBP

#### Women’s attitudes to their health

Women who took part in the interviews described being generally quite relaxed about their health. It was not something they worried about normally, although some said they were aware of their diet, exercise and general health during their pregnancy. Some of the women who were in their first pregnancy or included because of a higher BMI were somewhat surprised and resentful of being labelled at higher risk.

*“Despite being kind of singled out because of my age and my weight that I was high risk for preeclampsia, actually, my blood pressure was perfect all the way through” (04).*

But many women, in particular, those women who had previous experience of raised BP or pre-eclampsia, were understanding of the risks and felt happy to be included.

*“You don’t have to wait until the last minute so it was a great opportunity and I was very pleased to accept.” (10)*.

Participants were generally happy to engage with self-monitoring. It was described as convenient and a good opportunity to look after their health.

*“Having that opportunity to make sure everything is all right because you just don’t know what’s going on, do you, inside.” (13)*.

#### SMBP as reassuring not anxiety provoking

Women were asked if testing their BP during pregnancy made them feel more or less anxious. It was acknowledged that thinking about their BP could make them fearful as it might be raised when they measured it. But more commonly women said they were “reassured” by self-testing and regarded it as a good opportunity to keep an eye on themselves.

*“I think it was peace of mind for myself because pre-eclampsia can happen to me with, you know, given, you know, there’s no reason why it wouldn’t have, couldn’t have happened to me. So again, it was just the nice [um] nice reassurance.” (07).*

*“It makes you more anxious because it’s higher but it’s reassuring because you know you can do something about it. And that’s good because I didn’t feel any different.” (08).*

*“Less anxious, so much less anxious. Yeah so much less.” (13)*.

Women were asked what their partners and family thought about their participation in the study. While one respondent said that their partner expressed concern that it was raising her anxiety, most women described their partners as supportive of their self-monitoring.

Some women described feeling empowered, particularly if they had had a previous experience of raised BP or pre-eclampsia.

*“A good opportunity to take a little bit of control of your health” (06).*

*“Because I’m scientific and a little bit nosy about these things and, you know, I was looking forward to it for my own interest but I found it very reassuring and also very empowering.” (14)*.

Having access to their own readings also gave women the opportunity to reassure themselves if they felt unwell at any stage during their pregnancy. Participant 14 was in her first pregnancy and an older mother:

*“Because, at various points, I thought, I don’t feel very well [um] and I thought, I’ll just take my blood pressure and it was fine and it was exceptionally reassuring that to, be able to do that yourself and know that you’re okay.” (14)*.

Some participants experienced headaches and the monitoring was able to reassure them that they were not related to BP. Participant 11 had high BP with her first pregnancy and was a migraine sufferer, so she was happy that she could test herself if she started to feel unwell.

*“So I didn’t want to be worrying all the time that, “Oh my god, I’ve got a headache. Is it my blood pressure?” At least, with having the monitor at home, I could double check for myself and that’s really, what, why I wanted to take part.” (11)*.

Participant 05 took part in the study during her second pregnancy. She had pre-eclampsia during her first.

*“I was happy to do it and, like I said, there were times when I thought it was probably going to be beneficial as well for my own peace of mind. I kind of, there was a couple of times when I had a headache and it would have been something that I perhaps would have, eventually, gone to a GP about [um] to get it resolved and just knowing that I was going to be testing my blood pressure either that day or the following day. And it just reassured me a little bit that [um] that it would be all right.” (05).*

#### Understandings of the study, BP and risks of high BP in pregnancy

Participating women seemed to have a clear understanding of the aims of the trial. One woman was clearly aware that pre-eclampsia could develop rapidly and therefore felt it was good for women to measure their own BP:

“*In such a condition*, *every minute is important*” (03).

Some women had previous experience of pre-eclampsia so were aware of the risks. Others had just a vague understanding of the condition and its symptoms, but understood that it was important.

*“It increased my awareness of the potential conditions I could suffer from, and I thought that was a very positive thing.” (14)*.

While most understood that the trial was trying to improve the early detection of pre-eclampsia, some would have liked more information about the condition. Participant 14 wanted to understand what pre-eclampsia was and more about why it was important to do the work.

*I think it took me a while to actually understand what preeclampsia was. I think there could have been more information about what preeclampsia was as a condition and how it affected women and, therefore, why it was important to do this work. (14).*

Consistent with other qualitative research into the motivations behind trial participation [25] women described their motivations for agreeing to take part in the trial as two-fold; being able to help others (through research) and at the same time helping themselves. Women who had already experienced pre-eclampsia were especially keen to participate, seeing SMBP as a way of offering peace of mind and keeping themselves safe during their pregnancy.

*“I’d rather know my blood pressure than not know it. [um] And it was a way of keeping safe. It felt like that anyway to me.” (09).*

*“An opportunity to contribute my own little portion” (10)*.

Women said they found the timetable of the trial easy to understand and that it was clear to them what to do/who to call if their BP was raised.

#### The pregnancy and identifying raised BP

Most women remained well throughout their pregnancies, and for most self-monitoring did not detect any raised BP and therefore had no discernible effect on the course of their pregnancy. However raised BP was picked up in a small number of women.

Participant 01 took part in the study during her second pregnancy. She had developed gestational diabetes and gestational hypertension in her first pregnancy. Home BP monitoring identified raised BP after her son was born. She spoke to her midwife, who identified that she needed to stop taking the ibuprofen she had been prescribed, as it is not recommended after 30 weeks according to NICE guidelines.

*“It was picked up because I was doing the study that I, that they realised that my blood pressure was elevated and I actually needed to come off ibuprofen because it wasn’t safe for me to stay on it and I wouldn’t have known that.” (01).*

Participant 02 was pregnant with her first baby, and raised BP was detected from 12 weeks. She was hospitalised several times for BP observations and finally induced 3 weeks early. She was pleased she was self-monitoring her BP as she did not feel unwell even though her readings were high.

*“Yeah, sometimes it was a real shock. You’d just be laying there and they’d take your blood pressure and, yeah, it’d be really high.” (02).*

Participant 11 was a migraine sufferer, who had also had previous high BP. She had a couple of spikes of raised BP but it came down the next day. She was reassured by SMBP. Participant 08 was also relieved to be self-monitoring. At the end of her twin pregnancy her BP readings were high.

*“I found that really, really helpful because you can think, because I felt fine but if you’ve got high blood pressure, and I did it a couple of times extra just to make sure it wasn’t just that one time and it was a bit high but [um] there wasn’t there wasn’t anything to worry about, which was good, but it was really nice to have that reassurance.” (08).*

Participant 13 knew that she suffered from white coat hypertension (that is her BP was higher when measured in the clinic than otherwise), so she was very pleased to be able to take her BP herself.

*“From my perspective, I got a little machine to bring home and just from a, from an obsessive compulsive, you know, perspective of that blood pressure I was like, yeah, great, I get to do it three times a week and know that I’m okay and having that confidence that actually, my blood pressure was okay so if I went to the hospital, I could get out the book and say, it’s not, it’s not me. It’s because I’m here that it’s a little bit elevated.” (13)*.

Most women found their health professionals supportive of their self-monitoring and involvement in the study, but this was not always the case. Participant 09 was frustrated that she was forced to do 24-h ambulatory blood pressure monitoring (ABPM) after raised BP was picked up during a hospital visit. She felt that health professionals were dismissive of her readings.

*“And they wouldn’t listen to me that I’d done the trial, I had my folder there, I said, “Look, everything has been fine. I did it two days ago, it was fine. I did it this morning, it was fine.” But they wouldn’t listen to me”. (09).*

#### Practicalities of SMBP

There were varying experiences of fitting SMBP into daily life. Most women admitted to forgetting to take their BP at various stages, but described the different strategies they developed to remember and make it part of their daily routine. Participant 04 was an IT consultant who worked at home a lot. She set reminders on her computer calendar. Participant 05 found it hard with a young child and while still at work. Her husband did more of the early morning childcare and she set reminders on her phone for 7 am and 7 pm. Once women had established a routine for themselves they found it quite easy to remember and incorporate into daily life. But if that routine was disrupted (if they were away for work or on holiday, or after the birth of their baby), it was much harder to remember.

Not all women had signed up for the “Florence” text message system used in the study, which provided reminders and enabled women to submit their readings easily. Some really liked it; one commented she had “*baby brain*” (01) so appreciated the reminders.

*“Because even though you think taking your blood pressure three times a week, twice a day in those three times a week, you’d think, oh it was easy to remember but, when you’re juggling work and being pregnant and having a bit of pregnancy brain as well, it was just trying to remember all of these things and just a little nudge every morning was good to have it.” (07).*

Others opted out of the “Florence” text system, finding the messages irritating because they came at the wrong time.

*“Yeah, they were irritating because at a moment, I’d get three to four messages and I send my readings then then I got another message.” (03).*

Women had varying experiences of how long it took to take their BP. Some said it took a good 20 min each time, as it was important to rest before they took the readings. Others felt it only took a couple of minutes morning and evening. As participants were advised of the need to have five minutes rest before taking BP measurements, this may reflect a need for enhanced training.

The role and reassurance offered by the research midwife was a recurrent theme. Many women liked having access to another health professional and appreciated the extra support, reassurance and information that the research midwife was able to give them.

*“I feel like it is an extra gift for me to have extra help and extra advice and an extra person to look after you.” (03).*

*“Yeah, if anything she’s become more of a, she’s more of a a rock to me than my midwife was, so she was, I was always, we were always on the phone to each other. If ever I had any worries, I’d call [research midwife] rather than my midwife.” (07).*

Generally, women did not feel they were making extra appointments to take part in SMBP or the study. They appreciated being able to see the research midwife when they were at regular antenatal appointments so they did not need to take extra time off work.

Participant 03 was recently arrived from Pakistan and felt isolated when her husband was at work. She said that if the research midwife had not visited she would not have taken part, as it would have meant extra doctors’ visits that her husband would have to take time off for. However one woman (from Birmingham) felt that she missed out because on at least one of her appointments she saw the research midwife rather than the community midwife.

In terms of location, offering the opportunity to monitor at home was popular with women who knew they were affected by white coat hypertension. They were much happier doing it themselves.

*“I felt a lot more in control of the surroundings in which I was having it done. So I felt comfortable. I didn’t feel as if I was automatically going to get a high reading because I can I can honestly talk myself into a high blood pressure reading, I’m sure of it.” (13)*.

## Discussion

### Main findings

Our findings indicate that women participating in this qualitative study were willing to engage with SMBP, and that it could reduce anxiety around their health during their pregnancy. Although there was some variation in responses depending on whether women perceived themselves as high risk, many of the women interviewed felt reassured and empowered by SMBP, particularly if they had previous experience of pre-eclampsia. Women felt able to incorporate the monitoring into their weekly routines, with some adjustments, although this was more challenging in the post-partum period. The respondents felt that being involved in the study made them more aware of the risks of raised BP and pre-eclampsia.

### Strengths and limitations

This study included women with a range of ages, social circumstances and previous experiences including those with a previous history of pre-eclampsia and those simply at higher risk. However, it cannot be certain how far findings apply to women at higher risk of pre-eclampsia in general. We do not have insights from those who declined to be in the BuMP study at all, or women who withdrew, or from women who participated but did not respond to the interview invitation. Participants were generally White British, educated to degree level and may not be representative of the wider population. There was also an imbalance in numbers between women from Oxford and Birmingham, so the experiences from both sites were not equally represented. The supportive role of the research midwife in the Oxford sites was highlighted as a major factor in the success of the pilot in that location, but in Birmingham staffing changes were required due to ill health. However, this qualitative work with a small sample does provide valuable information to inform future research. The self-monitoring regime tested in the pilot study was feasible and acceptable to women, offering reassurance and empowerment. The qualitative component of the full trial will explore how far these findings may be generalisable to a more diverse range of women. This study included 15 women but given the relatively focused nature of the research questions, data saturation was reached (i.e. no new data on these themes emerge in the last few interviews) suggesting that the conclusions would not have been altered by a larger sample size. [23] Nonetheless, further research will enable us to assess whether further issues would be identified with a larger and more diverse sample.

### Interpretation

We are mindful that this research takes place amidst debates around the medicalisation of maternity care [26–29] and the complexities of articulating risk (for mother and baby) in contemporary childbirth practice [12]. However, the wider context of self-care and self-monitoring having the potential to enhance care, self-efficacy and empowerment should be acknowledged [30–33].

In terms of motivations and adherence to self-monitoring, the results from this study are similar to other trials of SMBP in the general population [34] and self-monitoring in other conditions such as asthma and diabetes [35–37]. However, this is the first qualitative study that authors are aware of regarding women’s experiences of self-monitoring their BP in pregnancy. Women in pregnancy are making decisions about both their own health and their baby’s health, which makes this a different experience from SMBP in the general population. Women with previous experience of pre-eclampsia are often not fully aware of their risks of hypertensive and cardiovascular problems in subsequent pregnancies or outside pregnancy [38].

Pregnancy is an anxious time for some women, even during a normal antenatal period and labour, in part generated by fears and uncertainty about the outcome [39]. Studies of women’s anxiety levels during labour and how they are affected by additional fetal monitoring [40] have shown that women can be reassured by active monitoring. Although the very use of fetal monitoring can imply birth is a risky process needing careful monitoring, women appeared reassured. It has been argued that asking women to monitor their BP will also raise their anxiety: a study of women who were offered screening for pre-eclampsia found that the screening process changed the way that women regarded their pregnancy, shifting it from a normal life event to something to worry about [15]. This was not born out by our findings, but our study focused on women identified with a higher risk of pre-eclampsia, according to NICE guidelines. As with patient experiences of SMBP outside of pregnancy, women gained knowledge, engagement and confidence in taking control of their own care [29, 34, 41, 42].

There is a potential concern that while SMBP might give women the opportunity to reassure themselves; this could be false in the case of pre-eclampsia with normal blood pressure. A potential learning point from this pilot study is that women should be given more education around pre-eclampsia and its symptoms and indeed several reported they would find this helpful. Several women described feeling reassured by normal BP in the context of feeling unwell but should be aware of the potential importance of other symptoms regardless of BP. These additional educational messages will be tested in a larger forthcoming trial, which will also assess outcomes with respect to timing of detection of pre-eclampsia.

As the availability and acceptability of SMBP increases [16], it is perhaps interesting to think of self-monitoring practices as adding to the cultural props that women can draw on during a time of anxiety. Machin and Scammell’s (1997) ethnographic work [43] on experiences of labour (using a ritual theory approach, exploring pregnancy and birth as a rite of passage), found that women relied upon and were reassured by the medical model of childbirth because that was the cultural tool offered to them. This could potentially be the case for self-monitoring and will be explored further in the qualitative component of the trial.

Using digital health solutions, such as mobile phone text monitoring, for the detection and management of medical conditions in pregnancy is a rapidly developing area of research. Technologies under investigation include SMBP, self-testing of blood sugar in women with gestational diabetes and self-testing for proteinuria [44–47]. Self-monitoring and self-testing combined with digital technology may detect problems earlier and engage patients in their care, providing rapid feedback to improve clinical management and allow electronic capture of data. This work shows that the development of home monitoring and testing for raised blood pressure is likely to be acceptable to many women at higher risk.

## Conclusion

This pilot work has found that SMBP during pregnancy is acceptable to women who are categorised as at higher risk of pre-eclampsia in pregnancy, and can be empowering and educative, but we still need to know more about women’s experiences of self-monitoring in pregnancy and the acceptability to a more socio-economically and ethnically diverse range of women. If self-monitoring becomes the norm and perhaps replaces professional monitoring in some circumstances, will it impact on the relationship and interactions women have with their health professionals during their pregnancies? Interventions can work in some places and settings, and not others, and have unintended consequences [48]. We plan to test the effectiveness of SMBP during pregnancy in a larger trial and also to explore the mechanisms for how SMBP might or might not work in conjunction with existing antenatal care, the barriers and facilitators to SMBP, and how bringing self-care into the home might impact on women’s experiences of pregnancy.

## Abbreviations

BP: Blood pressure
BuMP: Blood Pressure Self-Monitoring in Pregnancy study
NICE: The National Institute for Care and Excellence
SMBP: Self monitoring of blood pressure
